# Supplementary material for: Full Rescue of F508del-CFTR Processing and Function by CFTR Modulators Can Be Achieved by Removal of Two Regulatory Regions
Source: Int J Mol Sci. 2020 Jun 25;21(12):4524. doi: 10.3390/ijms21124524 (PMC7350234; doi:10.3390/ijms21124524)
Supplement: Supplementary file 1 [file ijms-21-04524-s001.zip › Uliyakina et al_Supplem Info_R1.pptx]

## Slide 1
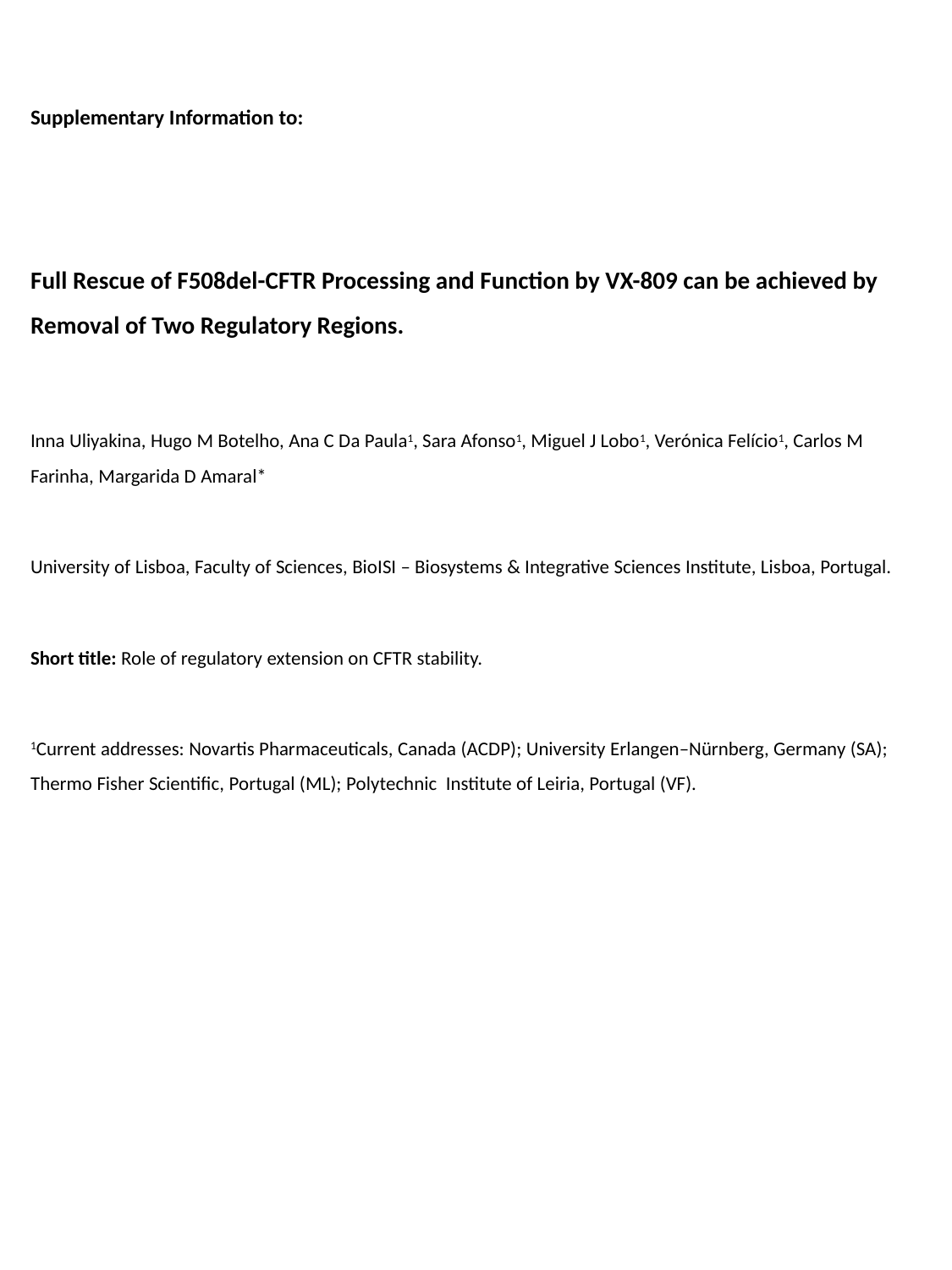

Supplementary Information to:
Full Rescue of F508del-CFTR Processing and Function by VX-809 can be achieved by Removal of Two Regulatory Regions.
Inna Uliyakina, Hugo M Botelho, Ana C Da Paula1, Sara Afonso1, Miguel J Lobo1, Verónica Felício1, Carlos M Farinha, Margarida D Amaral*
University of Lisboa, Faculty of Sciences, BioISI – Biosystems & Integrative Sciences Institute, Lisboa, Portugal.
Short title: Role of regulatory extension on CFTR stability.
1Current addresses: Novartis Pharmaceuticals, Canada (ACDP); University Erlangen–Nürnberg, Germany (SA); Thermo Fisher Scientific, Portugal (ML); Polytechnic Institute of Leiria, Portugal (VF).

## Slide 2
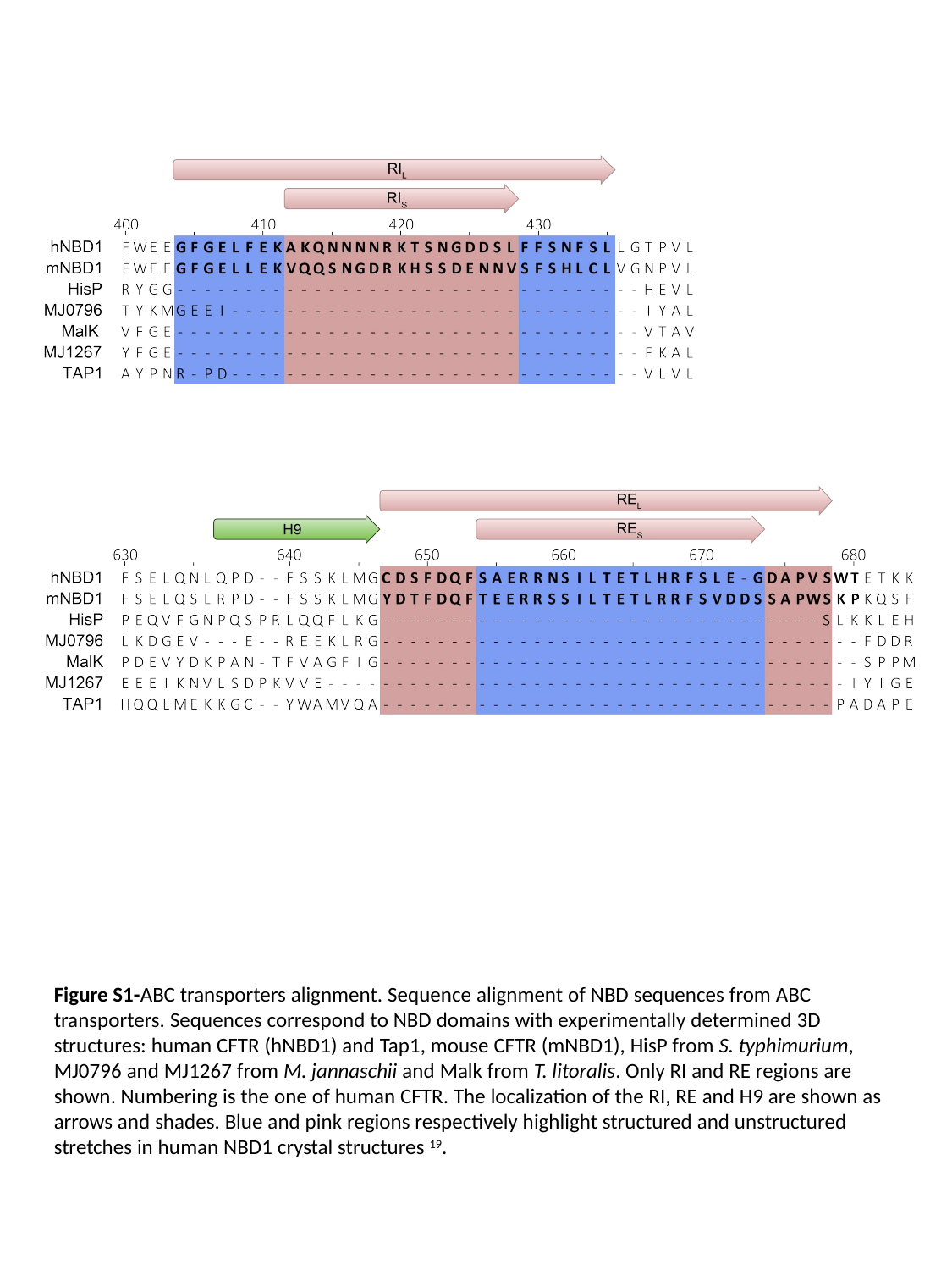

Figure S1-ABC transporters alignment. Sequence alignment of NBD sequences from ABC transporters. Sequences correspond to NBD domains with experimentally determined 3D structures: human CFTR (hNBD1) and Tap1, mouse CFTR (mNBD1), HisP from S. typhimurium, MJ0796 and MJ1267 from M. jannaschii and Malk from T. litoralis. Only RI and RE regions are shown. Numbering is the one of human CFTR. The localization of the RI, RE and H9 are shown as arrows and shades. Blue and pink regions respectively highlight structured and unstructured stretches in human NBD1 crystal structures 19.

## Slide 3
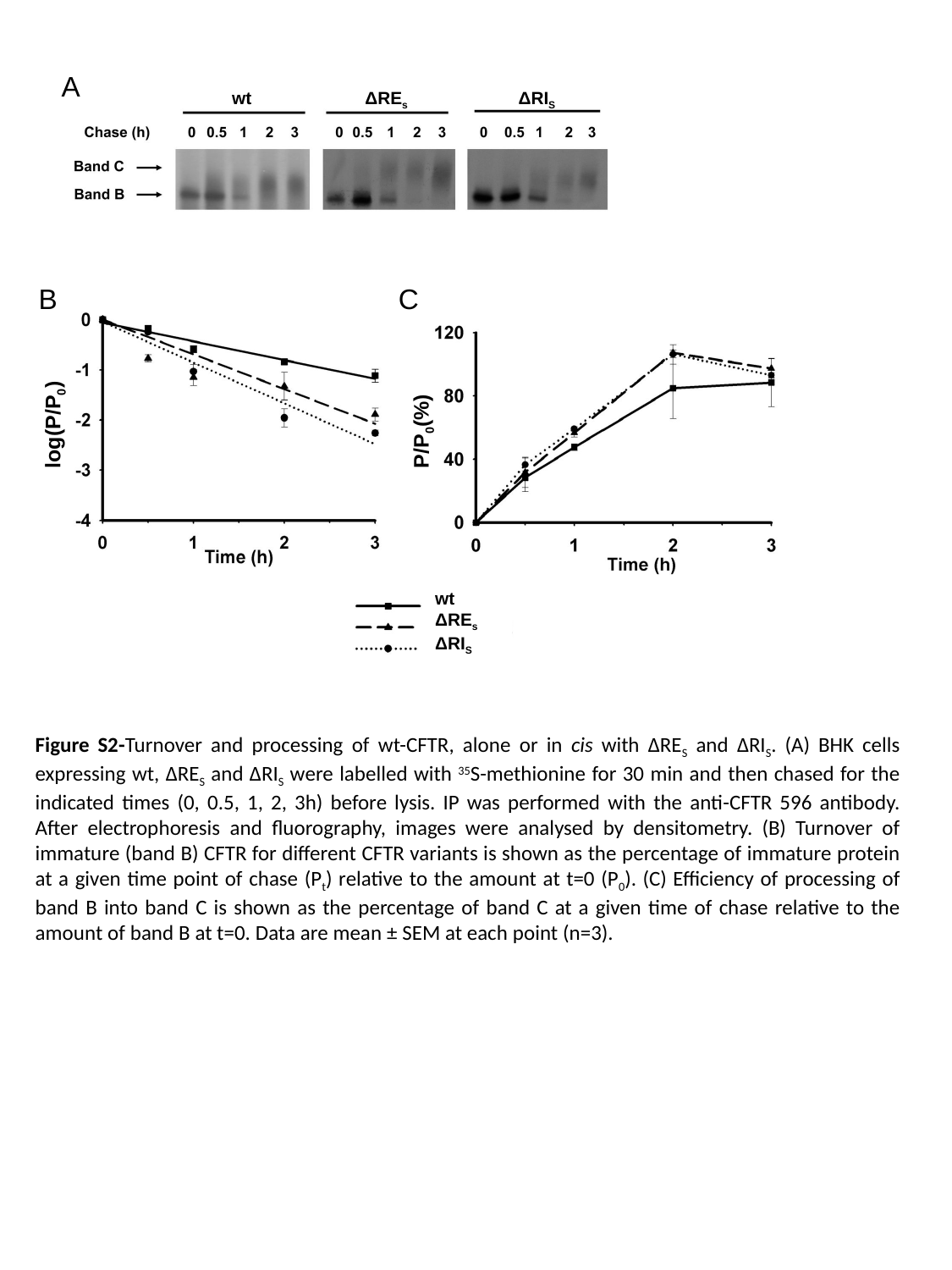

A
wt
ΔREs
ΔRIS
B
C
log(P/P0)
P/P0(%)
wt
ΔREs
ΔRIS
Figure S2-Turnover and processing of wt-CFTR, alone or in cis with ΔRES and ΔRIS. (A) BHK cells expressing wt, ΔRES and ΔRIS were labelled with 35S-methionine for 30 min and then chased for the indicated times (0, 0.5, 1, 2, 3h) before lysis. IP was performed with the anti-CFTR 596 antibody. After electrophoresis and fluorography, images were analysed by densitometry. (B) Turnover of immature (band B) CFTR for different CFTR variants is shown as the percentage of immature protein at a given time point of chase (Pt) relative to the amount at t=0 (P0). (C) Efficiency of processing of band B into band C is shown as the percentage of band C at a given time of chase relative to the amount of band B at t=0. Data are mean ± SEM at each point (n=3).

## Slide 4
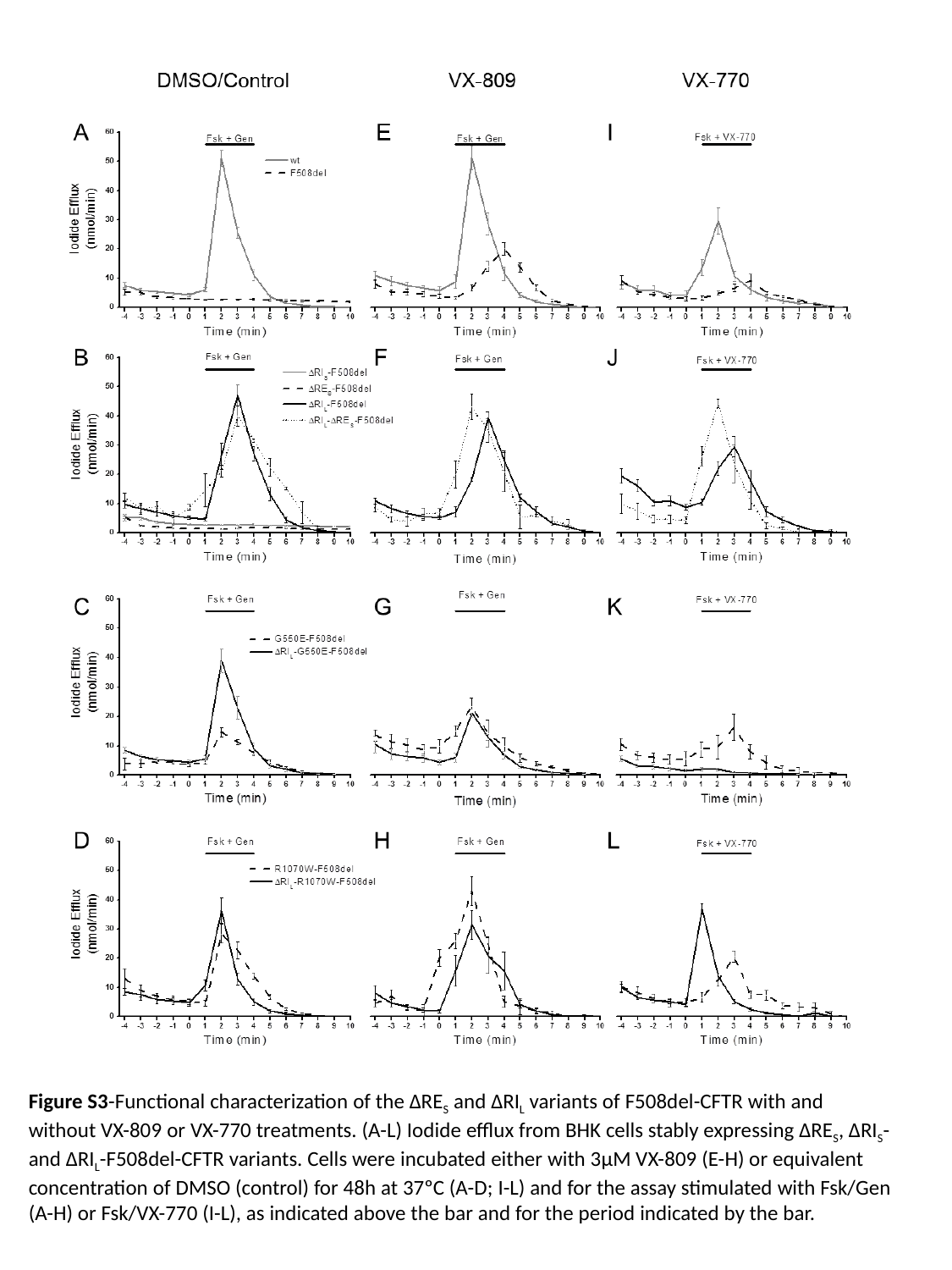

Figure S3-Functional characterization of the ΔRES and ΔRIL variants of F508del-CFTR with and without VX-809 or VX-770 treatments. (A-L) Iodide efflux from BHK cells stably expressing ΔRES, ΔRIS- and ΔRIL-F508del-CFTR variants. Cells were incubated either with 3µM VX-809 (E-H) or equivalent concentration of DMSO (control) for 48h at 37ºC (A-D; I-L) and for the assay stimulated with Fsk/Gen (A-H) or Fsk/VX-770 (I-L), as indicated above the bar and for the period indicated by the bar.

## Slide 5
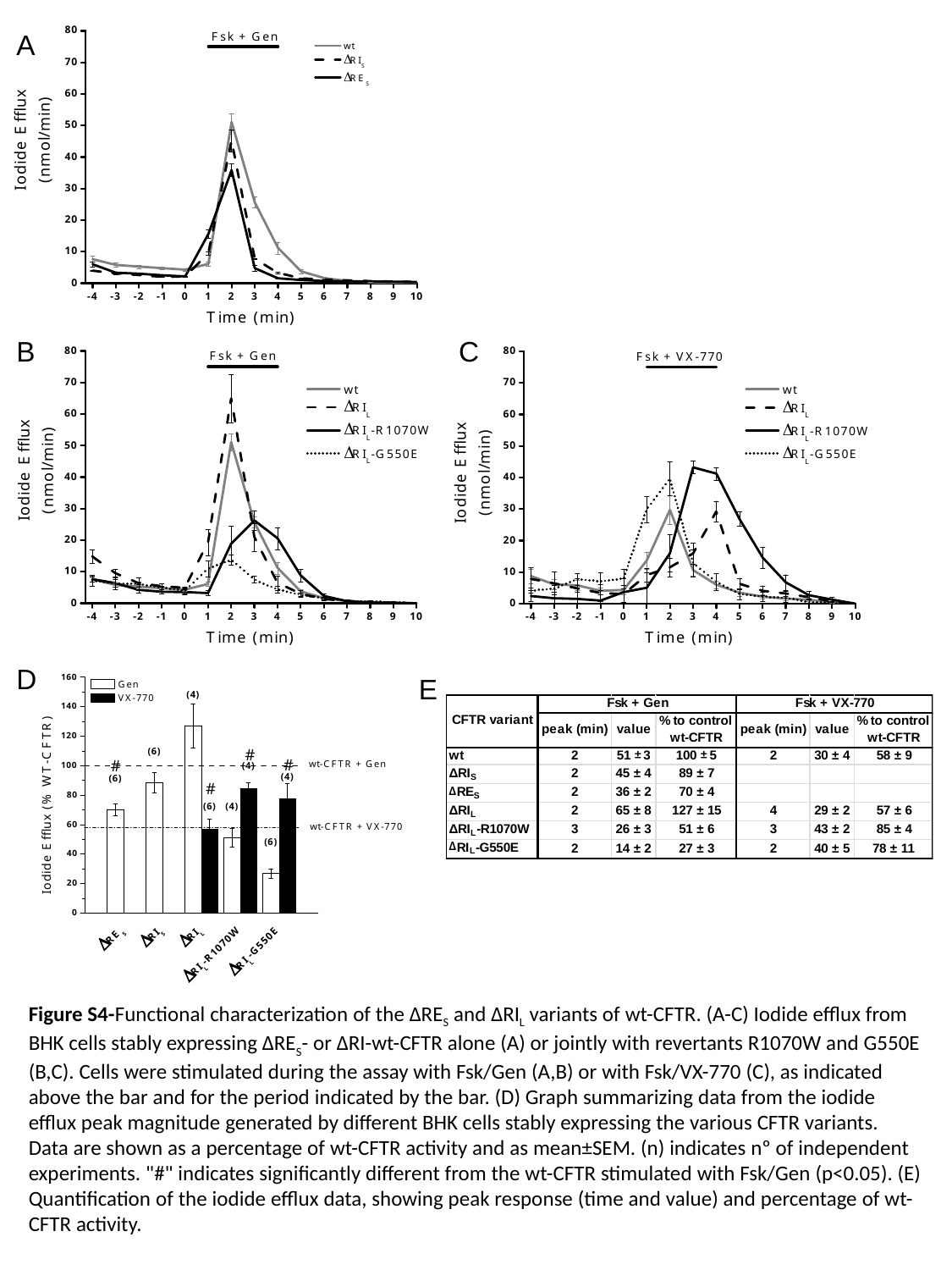

A
B
C
D
E
Figure S4-Functional characterization of the ΔRES and ΔRIL variants of wt-CFTR. (A-C) Iodide efflux from BHK cells stably expressing ΔRES- or ΔRI-wt-CFTR alone (A) or jointly with revertants R1070W and G550E (B,C). Cells were stimulated during the assay with Fsk/Gen (A,B) or with Fsk/VX-770 (C), as indicated above the bar and for the period indicated by the bar. (D) Graph summarizing data from the iodide efflux peak magnitude generated by different BHK cells stably expressing the various CFTR variants. Data are shown as a percentage of wt-CFTR activity and as mean±SEM. (n) indicates nº of independent experiments. "#" indicates significantly different from the wt-CFTR stimulated with Fsk/Gen (p<0.05). (E) Quantification of the iodide efflux data, showing peak response (time and value) and percentage of wt-CFTR activity.

## Slide 6
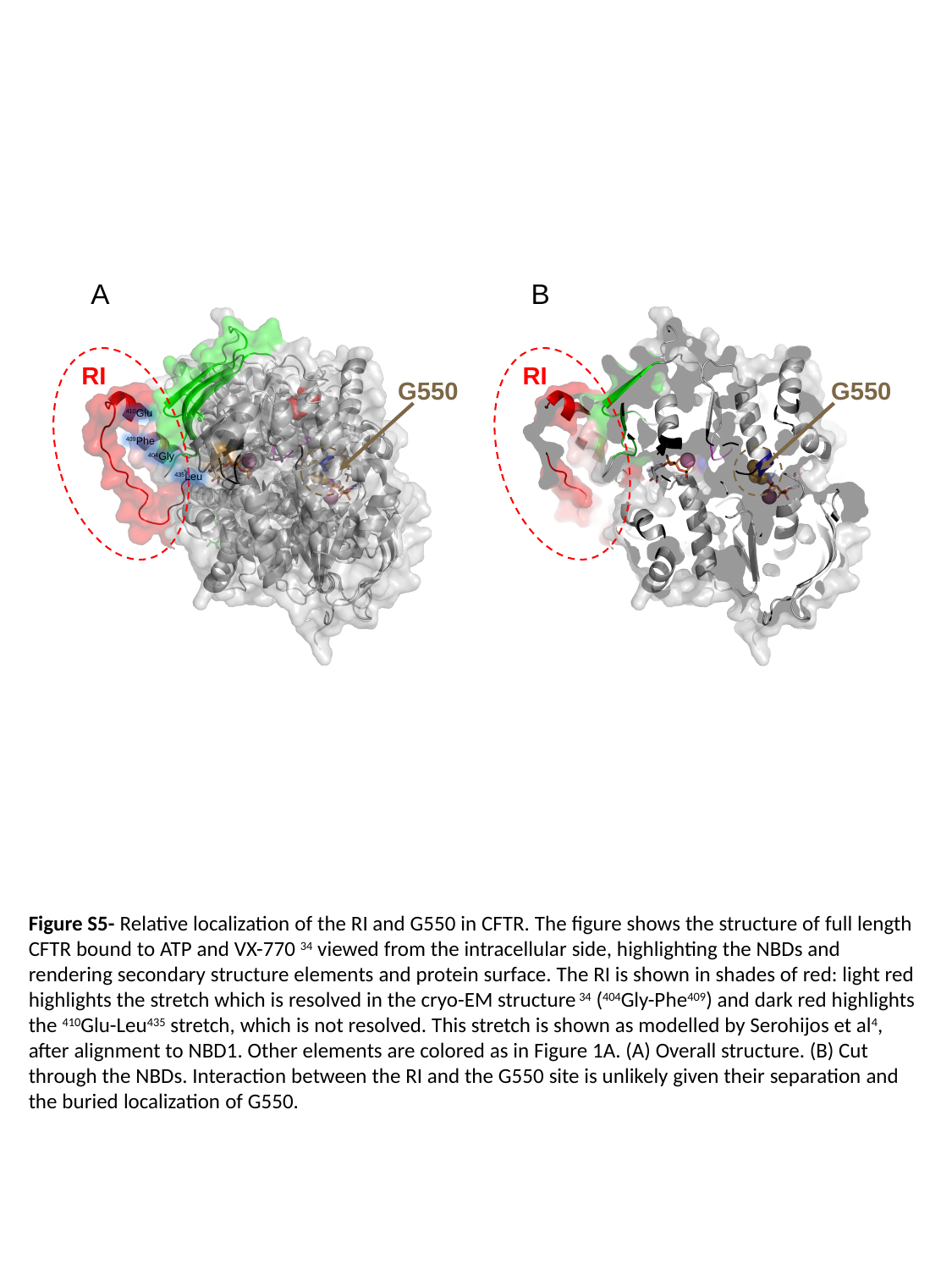

A
B
RI
RI
G550
G550
410Glu
409Phe
404Gly
435Leu
Figure S5- Relative localization of the RI and G550 in CFTR. The figure shows the structure of full length CFTR bound to ATP and VX-770 34 viewed from the intracellular side, highlighting the NBDs and rendering secondary structure elements and protein surface. The RI is shown in shades of red: light red highlights the stretch which is resolved in the cryo-EM structure 34 (404Gly-Phe409) and dark red highlights the 410Glu-Leu435 stretch, which is not resolved. This stretch is shown as modelled by Serohijos et al4, after alignment to NBD1. Other elements are colored as in Figure 1A. (A) Overall structure. (B) Cut through the NBDs. Interaction between the RI and the G550 site is unlikely given their separation and the buried localization of G550.
